# Genome-Wide Identification and Expression Profiling of Glutathione *S*-Transferase Gene Family in Foxtail Millet (*Setaria italica* L.)

**DOI:** 10.3390/plants12051138

**Published:** 2023-03-02

**Authors:** Linlin Wang, Hongbo Fu, Juan Zhao, Jiagang Wang, Shuqi Dong, Xiangyang Yuan, Xiaorui Li, Mingxun Chen

**Affiliations:** 1 State Key Laboratory of Sustainable Dryland Agriculture (in preparation), College of Agronomy, Shanxi Agricultural University, Taiyuan 030031, China; 2 Key Laboratory for Research and Utilization of Characteristic Biological Resources in Southern Yunnan, College of Biological and Agricultural Sciences, Honghe University, Mengzi 661100, China; 3 National Laboratory of Minor Crops Germplasm Innovation and Molecular Breeding (in preparation), Shanxi Agricultural University, Taiyuan 030031, China; 4 College of Agronomy, Northwest A&F University, Yangling 712100, China

**Keywords:** foxtail millet, glutathione *S*-transferase (GSTs), expression analysis, stress response

## Abstract

Glutathione *S*-transferases (GSTs) are a critical superfamily of multifunctional enzymes in plants. As a ligand or binding protein, GSTs regulate plant growth and development and detoxification. Foxtail millet (*Setaria italica* (L.) P. *Beauv*) could respond to abiotic stresses through a highly complex multi-gene regulatory network in which the GST family is also involved. However, *GST* genes have been scarcely studied in foxtail millet. Genome-wide identification and expression characteristics analysis of the foxtail millet GST gene family were conducted by biological information technology. The results showed that 73 *GST* genes (*SiGSTs*) were identified in the foxtail millet genome and were divided into seven classes. The chromosome localization results showed uneven distribution of *GSTs* on the seven chromosomes. There were 30 tandem duplication gene pairs belonging to 11 clusters. Only one pair of *SiGSTU1* and *SiGSTU23* were identified as fragment duplication genes. A total of ten conserved motifs were identified in the GST family of foxtail millet. The gene structure of *SiGSTs* is relatively conservative, but the number and length of exons of each gene are still different. The cis-acting elements in the promoter region of 73 *SiGST* genes showed that 94.5% of *SiGST* genes possessed defense and stress-responsive elements. The expression profiles of 37 *SiGST* genes covering 21 tissues suggested that most *SiGST* genes were expressed in multiple organs and were highly expressed in roots and leaves. By qPCR analysis, we found that 21 *SiGST* genes were responsive to abiotic stresses and abscisic acid (ABA). Taken together, this study provides a theoretical basis for identifying foxtail millet GST family information and improving their responses to different stresses.

## 1. Introduction

Glutathione *S*-transferases (GSTs), a superfamily of enzymes encoded by multiple genes and having multiple functions, are ubiquitous in animals, plants, and microorganisms. Glutathione binds to harmful heterologous substances or oxidation products through GSTs, thereby promoting the metabolism, compartmentalization, or elimination of such substances [1]. The classical reaction mode is that GSTs catalyze the binding of glutathione to various hydrophobic and electrophilic electronic groups to form soluble S-glutathionylated products [2]. Fourteen categories have been identified according to the amino acid sequence similarity, among which eight categories are extensive, including eight subclasses: tau (U type), phi (F type), lambda (L type), theta (T type), zeta (Z type), γ-subunit of translation elongation factor (EF1G), dehydroascorbate reductase (DHAR), and tetrachloro hydroquinone dehalogenase (TCHQD) [3,4,5]. Among these classes of GSTs, tau, phi, lambda, and TCHQD are endemic to plants, and the tau and phi classes are the most abundant GST types in plants [6,7]. Although the sequence homology of the GST gene is low (about 25%), it has been found, by studying the structure of a large number of GSTs proteins [8,9], that these proteins have highly conserved structural characteristics.

Since the first discovery of glutathione *S*-transferases in maize in the 1970s, GSTs have been identified as a multigene family [10], and GSTs have been found in many plants. Genome-wide analyses revealed that there are 55 *GST* genes in *Arabidopsis thaliana* [11,12], 79 in *Oryza sativa* [13,14], 330 in *Triticum aestivum* [15,16], 84 in *Hordeum vulgare* [17], 59 in *Gossypium Raimondi* [18], 141 in *Brassica napus* [19], and 52 in *Malus domestica* [20]. In addition, the *GST* genes were identified in these plants, laying the foundation for isolating new *GST* genes from other plants.

Many studies have shown that the plant *GST* gene family can regulate the adaptability of plants to various kinds of stresses through electrophilic substitution, detoxification, and peroxide scavenging [21,22,23,24]. The expression of this gene family is not just affected by drought [25], saline-alkali [26], low temperature [27], pathogen infection [28], herbicides [29], heavy metals [30], and other stresses. It is also subject to ABA [31], auxins (IAA), ethylene [32], salicylic acid (SA) [32], jasmonic acid (JA) [33] and other plant hormones.

The expression of *TaGSTU39* was significantly up-regulated throughout the treatment period under drought and salt stress treatments. *TaGSTU62* of wheat could be down-regulated by gibberellin (GA) and up-regulated by ABA [15]. In *A. thaliana*, overexpression of grape *GSTF13* could increase resistance to drought, salt, and methyl viologen stresses [34]. Multiple photoreceptors regulated the expression of *AtGSTU17* and various development of *A. thaliana* seeds, including hypocotyl elongation and anthocyanin accumulation [35]. *MdGSTF12* [20] and *MdGSTU12* [36] were strongly induced by aminolevulinic acid (ALA), and they play an essential role in ALA-induced anthocyanin accumulation in apples. *GmGSTU10* was induced explicitly by soybean mosaic virus and might have a highly efficient catalytic role in soybean [37].

A growing world population challenges global food and nutrition security [38]. In order to find suitable staple foods to overcome these difficult situations, millets are one of the potential candidates [39]. Foxtail millet is the oldest cultivated crop in the world, including China, and it is also the characteristic food crop in arid and semi-arid areas in northern China. Foxtail millet, as the main cultivated crop in dry green farming, has the characteristics of small genome size, short life cycle, self-pollination, drought resistance, and is able to grow in low fertility conditions, making it a model plant for studying C_4_ cereal crops [10,40,41,42]. However, there is no relevant report on the type, quantity, structure, and function of GST family in foxtail millet. Therefore, this study used genome and bioinformatics methods to analyze the *GST* gene family of foxtail millet transcription factors. A total of 73 *GST* genes were identified and divided into seven classes. The protein physicochemical properties, chromosomal distribution, gene structure, conserved motifs, cis-acting element, and gene expression of members of the GST family in foxtail millet were analyzed. We also exhibited the syntenic correlation between foxtail millet and *A. thaliana* genes. In addition, we investigated expression profiling of *GST* genes in different tissues and detected the expression of *GST* genes after various biotic and abiotic stresses in foxtail millet. Our study suggested that *GST* genes may play a role in regulating development and responding to various biotic and abiotic stresses and to ABA. Overall, this study provided a comprehensive identification of foxtail millet GST family members and provided a theoretical basis for further research on the functional analysis, gene editing, and genetic engineering of *GST* genes in foxtail millet.

## 2. Results

### 2.1. Identification of the Foxtail Millet GST Proteins and Analysis of Phylogenetic Relationship

**Figure 1:**
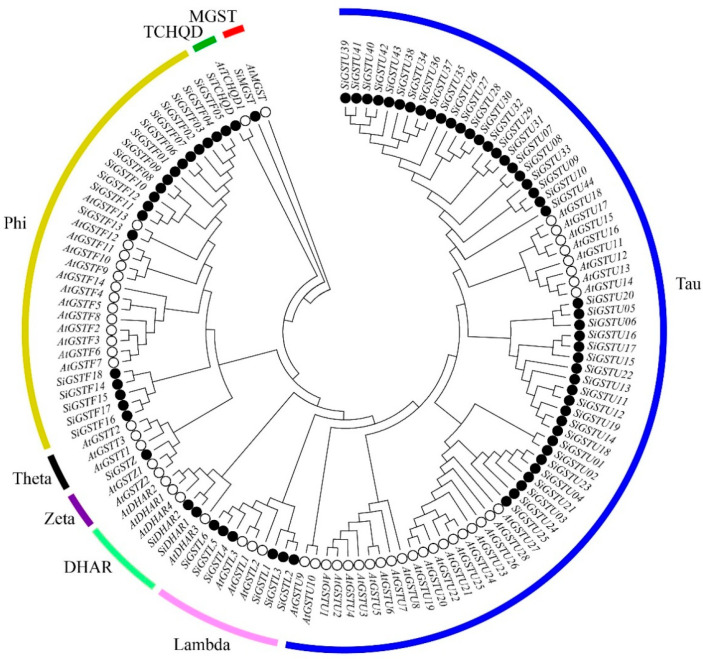
Phylogenetic tree of GST proteins among foxtail millet and *A. thaliana*. The GST protein sequences of 73 foxtail millet and 55 *A. thaliana* are divided into eight classes. Different subfamilies are marked as different colors.

The GST protein sequences of *A. thaliana* were searched against the protein sequences of foxtail millet to identify the GST proteins in foxtail millet. A total of 73 SiGST proteins were identified from the foxtail millet genome, based on the conserved GST-N and GST-C domains from these proteins (Figure S1). These GSTs were divided into seven distinct classes according to their conserved domains: tau, lambda, zeta, phi, DHAR, TCHQD, and MGST, and theta is absent in foxtail millet (Figure 1; Table S1). Among SiGST proteins, there are 44 tau proteins and 18 phi proteins, accounting for the vast majority, just as there are more tau and phi proteins in most plant GST families [43]. Next is lambda, which contains six members. The DHAR class had two members, and the zeta, TCHQD, and MGST classes had the least number, all of which had only one member.

The analysis of physicochemical property showed that the sequence length of SiGST proteins varied from 182 (*SiGSTF05*) to 320 (*SiGSTF18*) amino acid residues, and the molecular weight (MW) was 20,177.45 (*SiGSTF05*)—36,490.24 (*SiGSTF18*) Da. The isoelectric point (pI) values were changed from 4.74 (*SiGSTL1*) to 9.05 (*SiTCHQD*). The instability index of proteins ranged from 23.76 (*SiDHAR2)* to 60.81 (*SiGSTL5*), of which 31 were instability proteins with an instability index greater than 40. According to the correlation principle of the grand average of hydropathicity (GRAVY), the amphiphilic protein is between −0.5 and 0.5, the positive is hydrophobic protein, and the negative is hydrophilic protein. *SiGSTF14*, *SiGSTF18*, and *SiGSTU02* are hydrophilic proteins, and other proteins are amphotropic proteins, among which *SiGSTU08* has a maximum value of 0.185 and *SiGSTF18* has a minimum value of −1.085. The detailed data information for 73 SiGST protein sequences was tabulated (Table S2).

### 2.2. Chromosome Location and Gene Replication of SiGST Genes in Foxtail Millet

**Figure 2:**
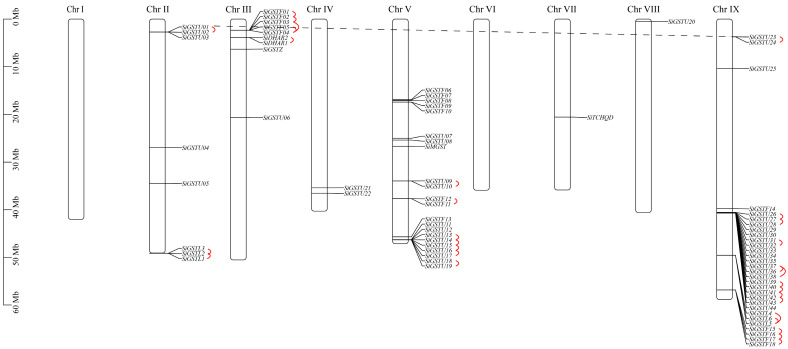
Chromosome mapping of *GST* genes in foxtail millet. A total of 30 tandem duplication gene pairs belonging to 11 clusters were present (highlighted by full red lines). Only one pair of gene segmental duplication event was found (highlighted by dashed black line).

The chromosomal localization of 73 *SiGST* genes in foxtail millet revealed that SiGSTs were unevenly distributed on seven chromosomes. A high-density region containing GSTs was found on chromosomes III, V, and IX (Figure 2; Table S3). Among them, chromosome IX with 30 *SiGST* genes included the most members, followed by 22 on chromosome V. Eight *SiGST* genes were distributed on chromosome II, and nine *SiGST* genes were distributed on chromosome III. Only two *SiGST* genes were distributed on chromosome IV, and both chromosomes VII and VIII with one *SiGST* gene contained the least members. There is no *SiGST* gene on chromosomes I and VI.

Segmental duplication and tandem duplication are considered to be two important factors for gene family expansion [44]. Among the 73 *SiGST* genes, a total of 30 gene pairs of 11 clusters were identified as the tandem duplication type (Figure 2). Among them, one pair of tandem duplication in DHAR class, four pairs in lambda class, eight pairs in phi class, and 17 pairs in tau class, indicating that a tandem duplication event contributed more to the expansion of the phi and tau classes. Only a pair of gene segmental duplication events (*SiGSTU1* and *SiGSTU23*) occurred in all classes on seven chromosomes. Further analysis of the evolution of *SiGST* genes revealed that there was no syntenic relationships between *SiGST* gene and *AtGST* gene (Figure S2).

### 2.3. Conserved Motif and Gene Structure Analysis of SiGSTs

**Figure 3:**
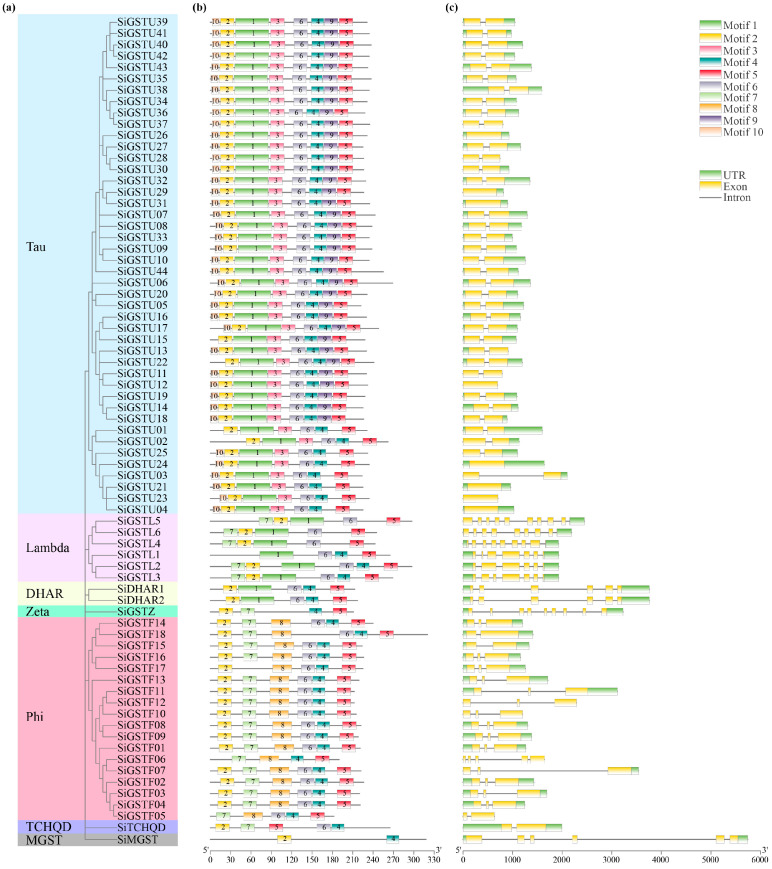
Gene structure and conserved motif of foxtail millet *GSTs*. (**a**) Neighbor-joining (NJ) phylogenetic tree based on SiGST protein sequences. (**b**) Conserved motif of *SiGSTs*. (**c**) Gene structure of *SiGSTs*.

In order to better demonstrate the diversity and similarity of the SiGST motifs, the conserved structure of amino acids in the foxtail millet GST family was analyzed using the MEME database (Figure 3a,b). The results showed that ten conserved motifs were identified in the foxtail millet GST family, and each conserved motif length ranged from 11 to 50 amino acids (Figure S3). Tau and phi have more members, with motifs 1, 2, 3, 4, 5, and 6 found in 44 tau protein sequences, and motifs 4, 5, 7, and 8 found in 18 phi protein sequences. There were 72 SiGST proteins that contained motif 5, while 70 SiGST proteins contained motifs 2, 4, and 6. In lambda classes, there were coexistent motifs. In addition, some class members had completely identical motifs, such as DHAR, which contains motifs 1, 2, 4, 5, and 6.

Gain or loss of introns can alter gene structure and play a crucial role in the evolution of gene families [45]. Gene structure analysis showed that *SiGSTU11*, *SiGSTU12*, *SiGSTU23*, *SiGSTU28*, *SiGSTU37*, *SiGSTF05*, *SiGSTF06*, *SiGSTF10*, and *SiGSTF12* did not contain upstream and downstream regulatory regions (UTR). *SiGSTU02*, *SiGSTU03*, *SiGSTU10*, *SiGSTU25*, *SiGSTU29*, *SiGSTU30*, *SiGSTL5*, and *SiGSTF07* did not contain the upstream regulatory region outside. *SiGSTU13* did not contain the downstream regulatory region outside. The other 55 members all have an upstream and downstream regulatory region (Figure 3c). At the same time, the gene sequence structure of the members of the GST family was analyzed, and the “exon-intron” structure diagram was obtained. Of these, most tau, phi, and TCHQD classes had one to three exons, while a phi member consisted of five exons. The DHAR and MGST classes exhibited six exons, while the zeta and lambda classes contained more exons than other classes (6–10 exons). The number of introns of the 73 *GST* genes in foxtail millet was less than ten, the most had nine, and the least had none. In addition, each member of the same subfamily has the same or similar gene structure. For example, most tau classes contained one intron; phi classes contained one to two introns, but *SiGSTF06* contained four introns; TCHQD classes contained one intron; DHAR and MGST classes contained five introns; and zeta classes contained eight introns.

### 2.4. Prediction of Cis-Acting Elements in Promoter Region of GSTs Gene Family Members in Foxtail Millet

**Figure 4:**
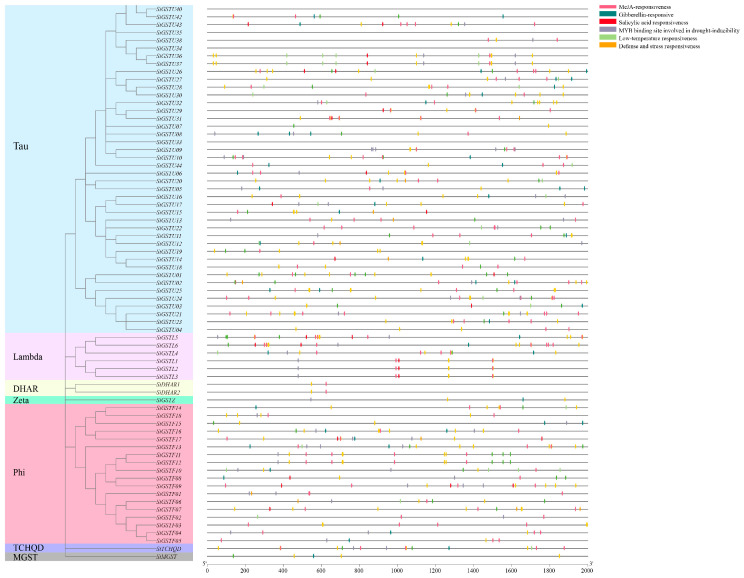
Predicted cis-acting element in *SiGST* promoter of foxtail millet.

Using PlantCARE to analyze the 5′-upstream promoter (2.0 kb) region of 73 *SiGSTs*, we found that the cis-acting elements in the promoter region of 73 *SiGST* genes mainly included 20 kinds, including defense and stress responsive elements involved in salt, drought, low-temperature and anaerobic, light responsive element, hormone-responsive element associated with IAA, ABA, Methyl Jasmonate, GA, SA, and other elements related to growth regulation and circadian control, including meristem expression element, cell cycle regulation element, endosperm expression element, seed-specific regulation element, root specific element and MYB (MER), MYBHv1 binding site (Figure 4 and Figure S4; Table S4). The promoter regions of 67 *SiGST* genes presented in defense and stress response elements, and the cis-acting elements of 67 *SiGST* gene promoters contained hormone responsive elements. In addition, more than 40 cis-acting elements were identified in the promoters of the *SiGSTF07*, *SiGSTF13*, *SiGSTU21*, and *SiTCHQD* transcripts. However, only two cis-elements were identified in the promoters of *SiGSTU39*.

### 2.5. Relative Expression Patterns (FPKM Value) of GST Genes in Different Tissues

**Figure 5:**
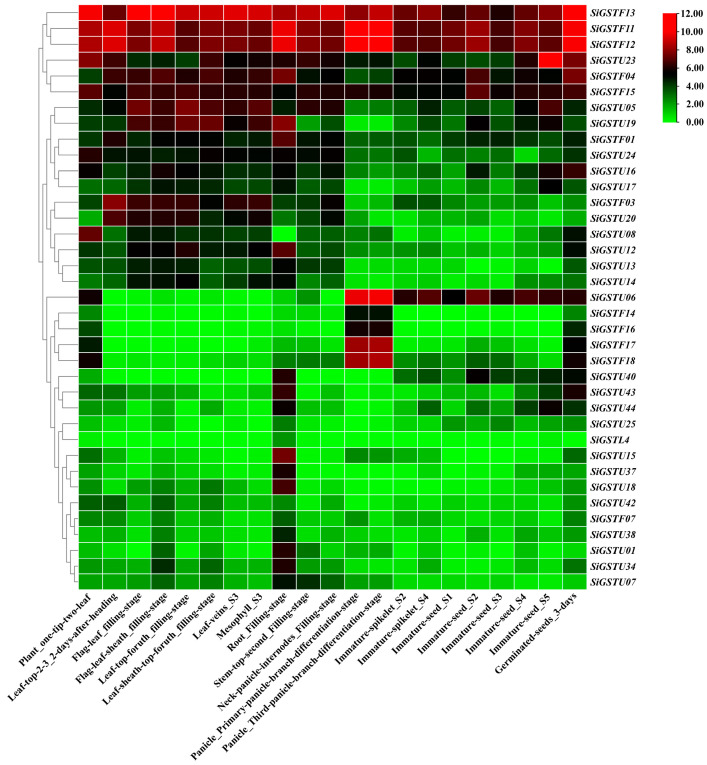
Relative expression patterns (FPKM value) of *GST* genes involved in 21 tissues of foxtail millet. The FPKM values of *SiGST* genes in different tissues were obtained from the *Setrari italica* Multiomics database (http://foxtail-millet.biocloud.net/home, accessed on 8 June 2022). The color bar represents log_2_ expression levels (FPKM), with red indicating high gene expression levels and green indicating low gene expression levels.

To explore the expression specificity of *GST* genes in foxtail millet at different tissues and developmental stages, we analyzed the expression profiles of 37 *SiGST* genes covering 21 tissues at different growth stages (Figure 5; Table S5). The results demonstrated partial differences in the expression of *SiGST* genes in different tissues of the same class. Additionally, most of the *SiGST* genes were expressed at higher levels in roots, which indicates that they may first play a role when the roots sense adversity. The transcription abundance of individual *SiGST* genes was low in all tissues and organs, such as *SiGSTF07,* which was weakly expressed in all tissues. However, some members had high transcript richness in all tissues and organs. *SiGSTF11*, *SiGSTF12*, and *SiGSTF13* were strongly expressed in all tissues. There were tissue-specific expression characteristics in different *SiGSTs* members, such as *SiGSTF01*, *SiGSTF03*, *SiGSTU05*, and *SiGSTU19*, which are highly expressed in the leaves and roots of foxtail millet during grain fillinfigure s1 stage. The expression of *SiGSTU06* in developing seeds and spikelets is relatively high, especially in panicle, which have the highest expression level. *SiGSTL4* was slightly or even not expressed in different tissues. In addition, the genes were differentially expressed in different tissues and organs at the same developmental stage. *SiGSTF11* and *SiGSTF12* were highly expressed in roots during the filling stage and *SiGSTF13* was highly expressed in neck-panicle-internodes during the filling stage. These results indicated that the relative expression pattern of *SiGST* genes in different tissues predicted its complex roles in foxtail millet growth and development.

### 2.6. Relative Expression Patterns of 21 SiGSTs under Abiotic Stresses and ABA Treatments in Foxtail Millet

**Figure 6:**
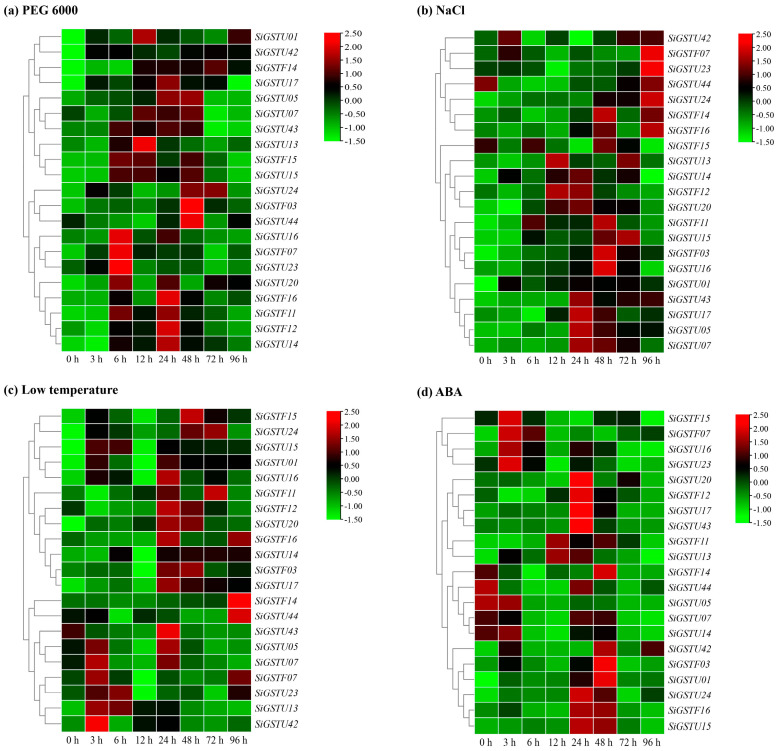
Relative expression patterns of 21 *SiGSTs* in leaves of foxtail millet under abiotic stresses and ABA treatments. Relative expression patterns of 21 *SiGST* genes were analyzed with qPCR under osmotic (20% PEG 6000) (**a**), salt (200 mM NaCl) (**b**), cold stress (4 °C temperature) (**c**), and 100 μM ABA (**d**). The relative expression levels of each gene were calculated by the 2^−∆∆Ct^ method. The heat map of SiGSTs expression condition was constructed by TBtools, expression value was standardized by Log2. The expression level of target gene at 0 h was used as control.

To understand the responses of *SiGST* genes to ABA and other abiotic stresses, we selected 21 genes to analyze their expression in foxtail millet treated with osmotic (20% PEG 6000), salt (200 mM NaCl), cold stress (4 °C temperature), and 100 μM ABA, respectively (Figure 6). In general, *SiGST* gene expression did not show consistent characteristics under stress and hormone treatments. Under osmotic stress treatment, the expression of *SiGSTF11*, *SiGSTF14*, *SiGSTU01*, *SiGSTU05*, *SiGSTU13*, *SiGSTU14*, *SiGSTU20*, *SiGSTU24*, and *SiGSTU42* were significantly up-regulated, and the expression level of *SiGSTU1* was remarkably up-regulated. Its expression peaked at 12 h, which was 56.5 times that of 0 h. The expression of *SiGSTF07*, *SiGSTU16*, *SiGSTU20*, and *SiGSTU23* peaked at 6 h; *SiGSTU13* at 12 h; *SiGSTF12*, *SiGSTF16*, *SiGSTU14*, and *SiGSTU17* at 24 h; and *SiGSTF03* and *SiGSTU44* at 48 h (Figure 6a).

Under salt stress treatment, the *SiGSTF03*, *SiGSTF11*, *SiGSTU01*, *SiGSTU15*, *SiGSTU24*, and *SiGSTU43* genes were induced more significantly, and the expression was higher than 0 h. The expression peak for *SiGSTF12* and *SiGSTU13* appeared at 12 h and up-regulated 7.2-fold and 6.7-fold compared to the control group. The expression levels of *SiGSTU05*, *SiGSTU07*, *SiGSTU14*, *SiGSTU17*, *SiGSTU20*, and *SiGSTU43* genes were the highest at 24 h, and the *SiGSTF03*, *SiGSTF11*, *SiGSTF14*, *SiGSTF15*, and *SiGSTU16* genes showed an expression peak at 48 h. The expression peak of *SiGSTF07*, *SiGSTF16*, *SiGSTU23*, and *SiGSTU24* appeared at 96 h, and *SiGSTU24* expression at 96 h, which is about 81 times that of 0 h (Figure 6b).

The expressions of *SiGSTF11*, *SiGSTF12*, *SiGSTF16*, and *SiGSTU43* were inhibited and lower than those of the control group in the early stage of 4 °C temperature treatment. However, *SiGSTU15*, *SiGSTU17*, *SiGSTU20*, and *SiGSTU24* expression levels were significantly higher than the control group during the whole 4 °C temperature treatment, and the *SiGSTU20* induced more remarkably. The expression levels of *SiGSTF12*, *SiGSTF16*, *SiGSTU05*, *SiGSTU16*, *SiGSTU17*, *SiGSTU20*, and *SiGSTU43* were significantly higher than the control group at 24 h. At 48 h, *SiGSTF12* and *SiGSTU20* were still significantly higher than in control group (Figure 6c).

Additionally, they could respond to ABA. For instance, the expression of *SiGSTU5*, *SiGSTU07*, and *SiGSTU44* were down-regulated by ABA; and the expression of *SiGSTF07*, *SiGSTU01*, *SiGSTU17*, *SiGSTU24*, and *SiGSTU42* could be up-regulated by ABA. The expression of *SiGSTF12*, *SiGSTU15*, *SiGSTU17*, *SiGSTU20*, *SiGSTU24*, and *SiGSTU43* peaked at 24 h. The expression peak for *SiGSTF03*, *SiGSTU01*, and *SiGSTU42* appeared at 48 h, up-regulated 16.1-fold, 19.2-fold, and 74.6-fold compared to the control group, respectively (Figure 6d).

## 3. Discussion

### 3.1. Identification and Analysis of GST Genes in Foxtail Millet

GSTs comprise a large and diverse gene family that is ubiquitous in a wide variety of organisms. Currently, the *GST* gene family has been confirmed to be involved in regulating the growth and development, stress resistance, and other processes of plants, such as *A. thaliana* [11,12] and rice [13], which has important biological significance. However, the identification and analysis of GST gene family in foxtail millet are still lacking. A total of 73 *SiGST* genes were identified from the foxtail millet genome, which were divided into seven categories. Among them, tau and phi contain the most SiGST family members, with 44 and 18 *SiGST* genes, respectively, which are the same as the findings of other plants such as soybean [46], rice [13], and pepper [47]. Tau and phi, known as plant-specific GSTs, had the most members and abundant content compared with other subfamilies [47]. A possible reason is that, compared with animals, in addition to detoxification, GST in higher plants also has unique functions such as transporting flavonoids, and regulating plant growth and development [30,48].

Gene structure is of great significance to the study of gene evolution [49]. The rearrangements and fusions of different chromosomal fragments may result in the gain or loss of an exon or intron, which play an important role in the diversification of multi-gene families [45]. We found that the number of *GST* exons is generally conserved within the same class in foxtail millet. GSTUs had one or two exons, GSTFs had two or three exons (except for *SiGSTF06*), DHAR and MGST had six exons, zeta had nine exons, and TCHQDs had two exons (Figure 3c). Similarly, the structural characteristics of *GST* genes were conserved in wheat, apple, and melon [15,20,50]. In addition, previous studies have shown that the earliest gene had the fewest number of introns, and as the replication progressed, the number of gene members continued to increase, and the number of introns gradually increased. It is pointed out that some of the origins of introns in the gene structure exist within themselves, and some are generated or increased with the insertion of transposons during gene replication [51,52]. In this study, through the analysis of the structure and conserved motifs of GST, it was found that most of the tau and phi classes contained one intron, the DHAR and MGST classes contained five introns, and the zeta class contained eight introns (Figure 3a,b). Therefore, according to the number of members of each subfamily and the number of introns in the gene, it is speculated that tau and phi may first appear in the GST family of foxtail millet. This was similar to the structure of GST family genes reported in wheat, apple, and radish [15,20,53].

The expansion of plant gene families is mainly achieved through different gene duplication methods (including fragment duplication, cascade duplication, gene transfer, and genome duplication, etc.) [18,54]. In addition, the expansion of GST family genes in plants was mainly completed by the tandem duplication of genes of tau and phi classes, making plant-specific tau and phi the two classes with the most members [6,47,55]. GST protein sequences of foxtail millet were compared, and the phylogenetic tree was constructed. It was divided into eight large groups (tau, phi, lambda, zeta, theta, DHAR, TCHQD, and MGST) (Figure 1). There were 28 *A. thaliana* genes in tau, accounting for 50.9% of the *A. thaliana* GST family. However, there were 44 members of the foxtail millet GST family in tau, accounting for 60.2%. This amplification in tau may be related to gene duplication during evolution. Indeed, we found that only one pair of segment duplication genes were in foxtail millet, and 30 pairs of genes were tandem duplications, and tandem duplications were more frequent than segment duplications (Figure 2). Similar phenomena have been observed in rice [13], apple [20], and *Capsella rubella* [56]. Gene duplication not only expands the content of the genome, but is also important for generating new gene functions, thereby enabling organisms to further adapt to complex environments [44]. Collinearity analysis indicated that strong selection pressure was subjected to during the evolution of *GST* gene family in foxtail millet.

### 3.2. The Expressions of SiGST Genes in Foxtail Millet

Tissue expression profiling analysis of *SiGST* genes indicated that most *SiGST* genes were expressed in multiple organs and highly expressed in roots and leaves, indicating that most *SiGST* genes may play a role in roots and leaves (Figure 5). We also found functional differentiation of *GSTs* in foxtail millet, and *SiGSTs* were expressed differently in different tissue parts of foxtail millet. For example, *SiGSTF03* was highly expressed in parietal leaf at heading stage. *SiGSTU06*, *SiGSTF17*, and *SiGSTF18* were highly expressed in panicle_primary-panicle-branch-differentiation-stage and panicle_third-panicle-branch-differentiation-stage. *SiGSTL4*, *SiGSTU15*, and *SiGSTU18* were specifically expressed in the underground part of foxtail millet. In some tandem repeat pairs, the expression levels of the two genes differed, suggesting that the retention of gene duplication may be related to the process of tissue expression differences [57,58]. For example, *SiGSTF15* was highly expressed in 21 different tissues, while *SiGSTF07* was less expressed.

Plant growth and development are affected by various abiotic stresses, which activate the molecular mechanisms of plants to adapt to adverse conditions [59]. Drought, high salinity, and extreme temperatures limit the geographical distribution of plants because they cause dehydration and, eventually, cell death [53]. The *GSTs* promoter region of foxtail millet contains a large number of light response elements and stress response elements (low temperature, drought, stress defense, and stress), and the cis-acting elements in promoter regions of *SiGST* genes were found to be involved in the response to diverse biotic and abiotic stresses, as well as hormones (Figure 4 and Figure S4; Table S4). The 94.5% of *SiGST* genes possessed defense and stress responsive elements, and only six of them had no hormones responsive elements.

Previous studies have shown that the GST gene family is induced by abiotic stresses and hormones. For example, *MdGSTF12* [20] and *MdGSTU12* [36] were strongly induced by ALA. *TaGSTU62* could be induced by osmotic stress, salt stress, and ABA [15]. However, *OsGSTU4* could only be induced by salt stress [60]. The expression profiles of 21 *SiGST* genes under three abiotic stresses and one hormone (osmotic, salt, cold stress, and ABA) were analyzed by qPCR, showing that *SiGST* genes could be induced by abiotic stresses and ABA, and they might play a key role in abiotic stress response through corresponding hormone-dependent pathways (Figure 6). This study found that the *SiGSTU17*, *SiGSTU24*, and *SiGSTF03* could be induced by abiotic stresses and ABA. Although these results indirectly proved that *SiGST* genes are involved in the response of Jingu 21 to stress, further analysis is still needed to confirm its ability to resist stresses.

## 4. Materials and Methods

### 4.1. GST Gene Identification, Phylogenetic Analysis and Physicochemical Properties of Foxtail Millet

To identify the SiGST proteins, the *Setaria italica* genome data were downloaded from the NCBI database (https://www.ncbi.nlm.nih.gov/genome/?term=Setaria+italica+, accessed on 14 August 2021). HMMER 3.0 software was used to identify *SiGST* genes, and the *GST* gene domain sequence (PF02798 and PF00043) was downloaded from the Pfam database (https://pfam.xfam.org/, accessed on 7 June 2022). The National Center for Biotechnology Information conserved domain database (www.ncbi.nlm.nih.gov/Structure/cdd/wrpsb.cgi, accessed on 7 June 2022) was used to detect GST domains which were then mapped to the conserved domain. Neighbor-joining (NJ) phylogenetic tree was constructed using MEGA 7 software by the neighbor-joining method (bootstraps = 1000). The GST protein sequences of *A. thaliana* (55 numbers) were downloaded from The *Arabidopsis* Information Resource (TAIR, http://www.arabidopsis.org, accessed on 27 October 2020). Length of the protein sequence, protein molecular weight (MW), genomic position, isoelectric point (pI), instability index, and aliphatic index were predicted using ExPASy-ProParam (https://web.expasy.org/protparam/, accessed on 9 June 2022).

### 4.2. Distribution and Duplication Analysis of SiGSTs

TBtools was used to display the distribution of *GST* genes on *Setaria italica* chromosomes. Two or more *GSTs* separated by no more than three genes on the chromosome are called *GST* gene clusters. The gene sequences were aligned using BLASTp to determine the form of gene replication with an e-value of 1 × 10^−1^.

### 4.3. Gene Structure, Motif Compositions, and Gene Synteny of SiGST Genes

The gene structure map was produced, and an intron-exon map was compiled based on the *S. italica* genome annotation information (v 2.0). The MEME database (http://meme-suite.org/tools/meme, accessed on 9 June 2022) was used to conduct protein motif analysis. Chromosomal positions of *SiGST* genes were analyzed, and MCScanX was used to detect collinear regions between *SiGST* genes, as well as collinear blocks of *SiGST* genes with *A. thaliana* genes. The *A. thaliana* genome data were downloaded from the NCBI database. All above were visualized using TBtools [61].

### 4.4. Prediction of Cis-Acting Elements in the Promoter of SiGST Genes

Using the genome sequence of *SiGSTs* obtained in the phytozome database, the 2.0 kb DNA sequence upstream of *SiGST* genes was submitted to the PlantCARE database (http://bioinformatics.psb.ugent.be/webtools/plantcare/html/, accessed on 17 June 2022) to predict cis-acting element. Promoter cis-acting regulatory element predictions were performed and visualized in TBtools.

### 4.5. Analysis of the Expression Pattern of SiGST Genes in Different Tissues

The FPKM values of *SiGST* genes, in the Multi-omics Database for *Setrari italica* (http://foxtail-millet.biocloud.net/home, accessed on 8 June 2022) in different tissues such as roots, stems, leaves, and flowers were extracted, and the TBtools software was used to draw gene expression heat maps for visualization.

### 4.6. Plant Materials and Treatments

The foxtail millet seeds of Jingu 21 were in a germination box with a temperature of 26 °C, a relative humidity of 65%, and a light cycle of 16 h/8 h. Two days later, the seedlings were transplanted into a plastic container with a modified half-strength Hoagland nutrient medium for further cultivation. For osmotic stress, salt stress, and ABA treatments, the 12-day-old seedlings were allowed to grow in 1/2 Hoagland solution containing 20% PEG 6000, 200 mM NaCl, and 100 μM ABA, respectively. For cold stress treatment, the 12-day-old seedlings were placed in a low temperature (4 °C) incubator to grow. The seedlings in 1/2 Hoagland solution without treatment at 26 ° C were regarded as controls. The leaves of the seedlings were sampled at 0 h, 3 h, 6 h, 12 h, 24 h, 48 h, 72 h, and 96 h after 20% PEG 6000, 200 mM NaCl, and 100 μM ABA treatment. All samples were immediately frozen in liquid nitrogen and stored in a −80 °C refrigerator.

### 4.7. RNA Isolation and qPCR

The acquisition of RNA from leaves was accomplished with TRIzol kit (Accurate Biology, Changsha, China), cDNA was synthesized using reverse transcription kit (Accurate Biology, Changsha, China), and real-time PCR was performed with SYBR Green dye method (Accurate Biology, Changsha, China). PCR primers were designed using Primer Premier 5 software (Table S6). The qRT-PCR reaction was performed in a Bio-Rad CFX system, a 20 µL reaction system containing 10 µL 2× SYBR Green *Pro Taq HS* premix, 2 µL cDNA, 0.4 µL each of forward primer and reverse primer, 7.2 µL RNase free water. The following cycling conditions were used: 95 °C for 30 s, followed by 40 cycles of 95 °C for 5 s, and 60 °C for 30 s. The *SiActin* (*SETIT_026509mg*) was used as internal standard, and the expression levels of each gene were calculated by the 2^−ΔΔCt^ method. The heat map of SiGSTs expression condition was constructed by TBtools, expression value was standardized by Log2. Each independent experiment was repeated at least three times.

## 5. Conclusions

Through the appraisal of foxtail millet *SiGST* gene families, system evolution, and expression analysis, identified 73 millet *SiGST* genes, and the chromosomal location, the physicochemical properties, gene structure, and conservative structure domain analysis, this study forecast the *SiGSTs* promoter segment in response to ABA treatment, several abiotic stresses, and developmental stages. The expression profile spectrum analyses of *SiGST* genes showed that most of the *SiGST* genes were highly expressed in the roots and leaves. The qPCR analyses of 21 *SiGST* genes confirmed that *SiGST* genes were widely involved in stress and hormone responses such as drought, salt, low temperature, and ABA. These results provide a reference for further research on the gene function of foxtail millet molecular breeding and mining potential genetic resources.

## Data Availability

All GST protein sequences are provided in Table S1.

## Supplementary Materials

The following supporting information can be downloaded at: https://www.mdpi.com/article/10.3390/plants12051138/s1, Figure S1: Conserved domain of foxtail millet *GSTs*. Figure S2: Collinearity analysis of *GSTs* in *Setaria italica* and *Arabidopsis thaliana.* Figure S3: The putative motifs of SiGST proteins in foxtail millet. Figure S4: Predicted cis-acting element in *SiGST* promoter of foxtail millet. Table S1: GST protein sequences of foxtail millet and *A. thaliana* used to construct the phylogenetic tree. Table S2: The detailed information of 73 *SiGST* genes, including gene name, accession number, number of amino acids, MW, chromosomal location, theoretical PI, instability index, aliphatic index, and grand average of hydropathicity. Table S3: The distributions of *SiGST* class members on foxtail millet chromosomes. Table S4: Putative cis-acting elements identified in the promoter regions of *SiGSTs*. Table S5: The expression levels (FPKM value) of *SiGST* genes involved in 21 tissues. Table S6: Primers used for qPCR.
