# Supplementary material for: Genome-Wide Identification and Expression Profiling of Glutathione S-Transferase Gene Family in Foxtail Millet (Setaria italica L.)
Source: Plants (Basel). 2023 Mar 2;12(5):1138. doi: 10.3390/plants12051138 (PMC10005783; doi:10.3390/plants12051138)
Supplement: Supplementary file 1 [file plants-12-01138-s001.zip › Figure S3. The putative motifs of SiGST proteins in foxtail millet.pdf]

Motif 1

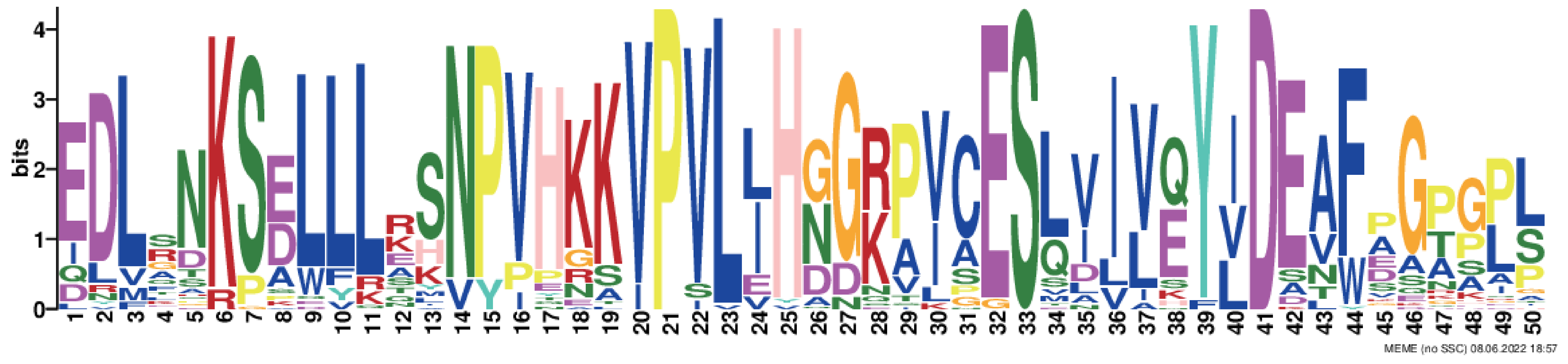

Motif 2

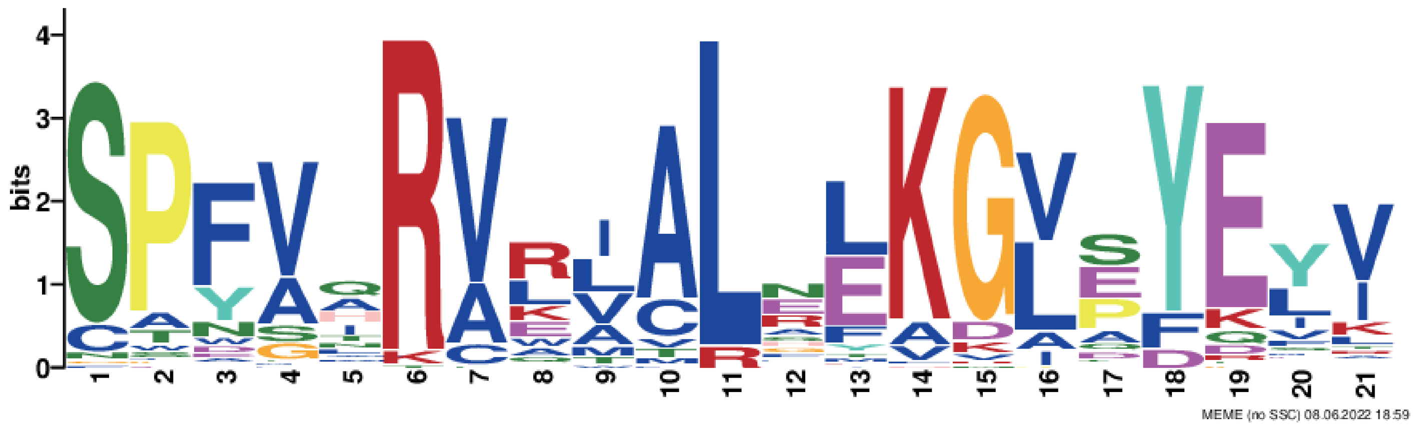

Motif 3

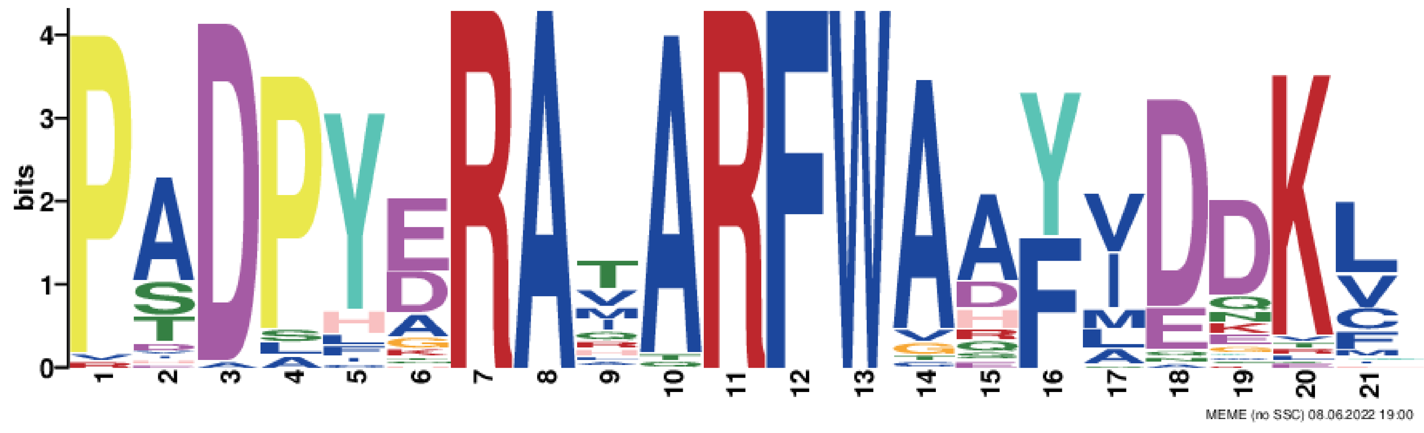

Motif 4

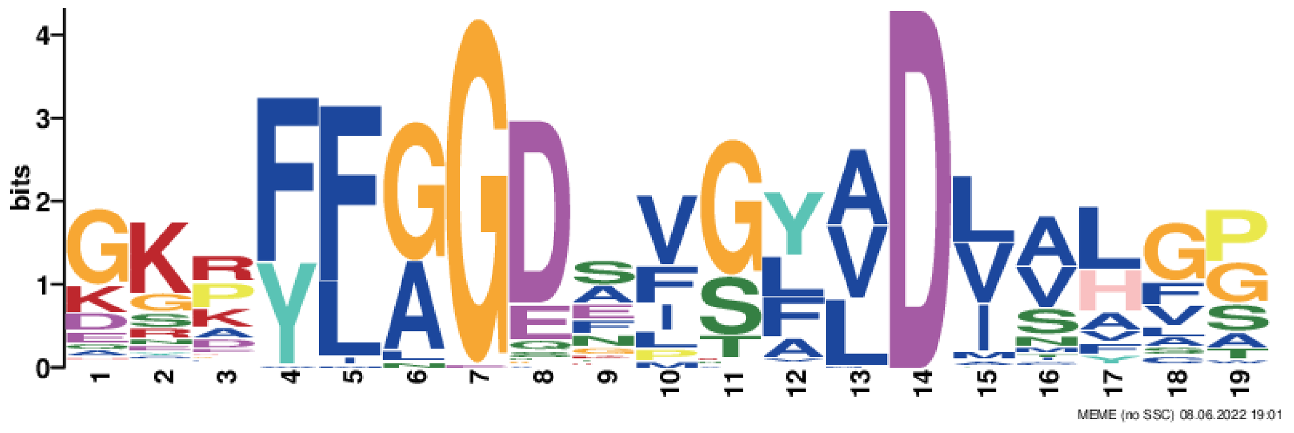

Motif 5

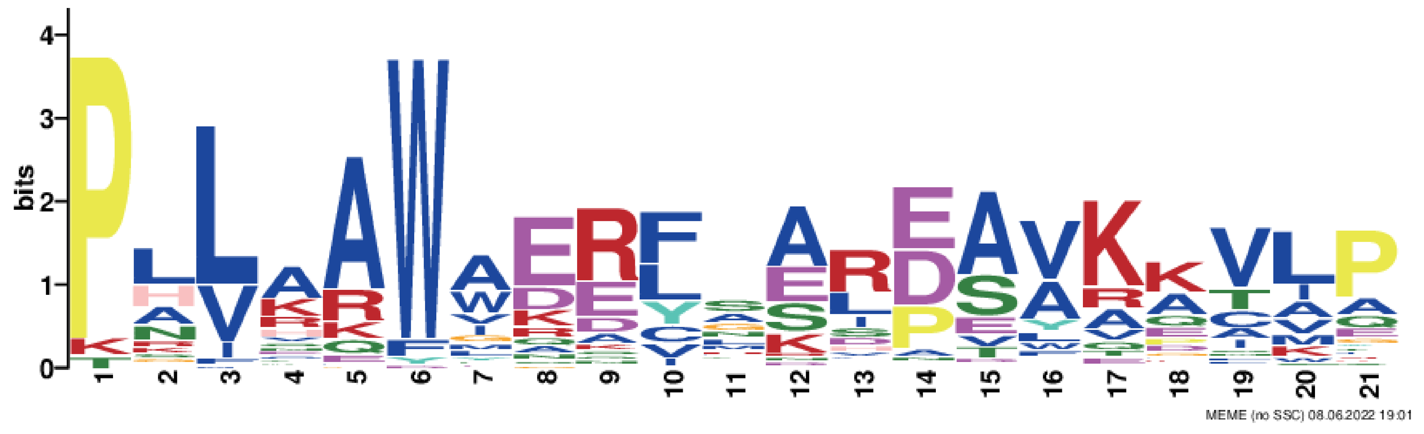

Motif 6

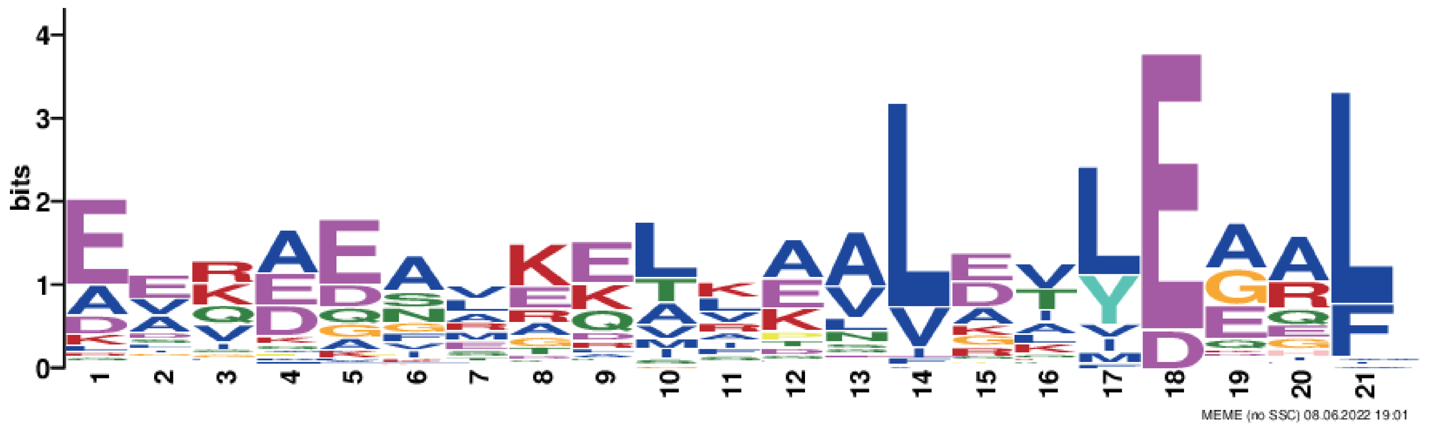

Motif 7

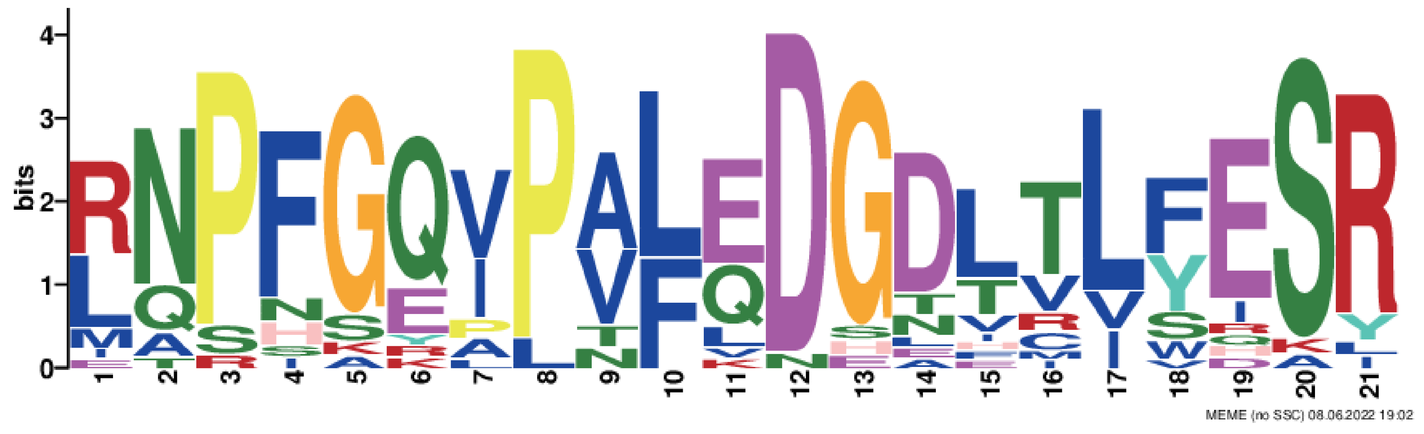

Motif 8

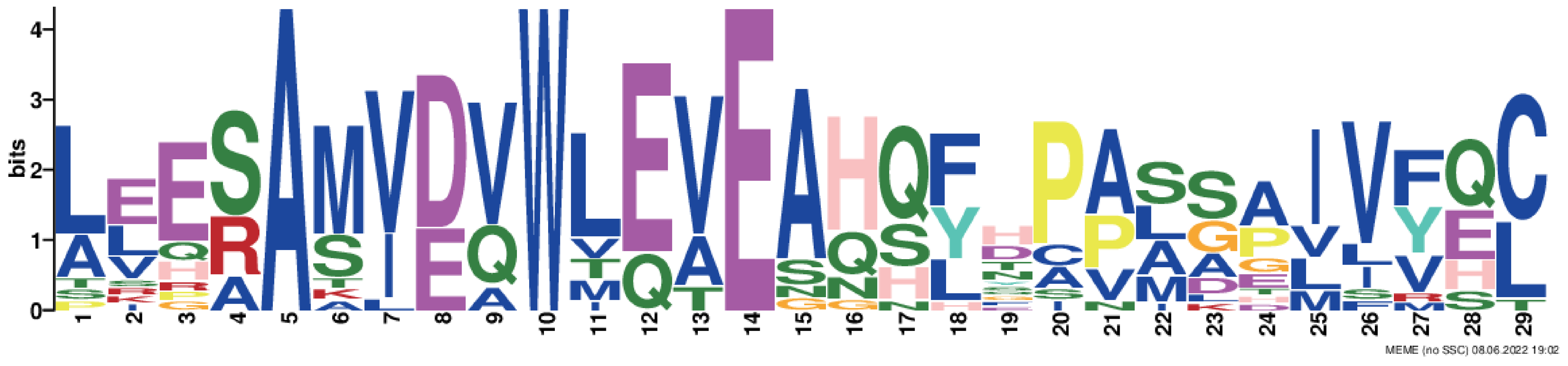

Motif 9

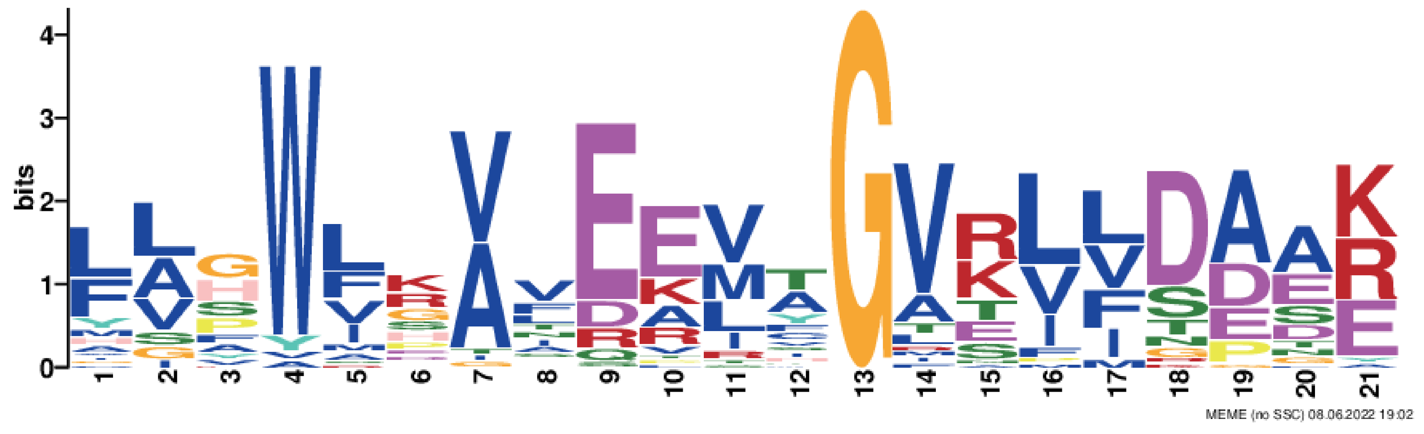

Motif 10

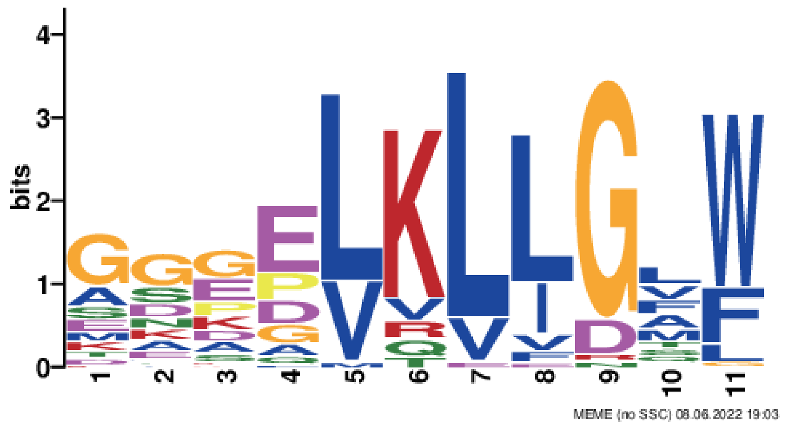

Figure S3. The putative motifs of SiGST proteins in foxtail millet.
