# Supplementary figures and images for: Genome-Wide Identification and Expression Profiling of Glutathione S-Transferase Gene Family in Foxtail Millet (Setaria italica L.)
Source: Plants (Basel). 2023 Mar 2;12(5):1138. doi: 10.3390/plants12051138 (PMC10005783; doi:10.3390/plants12051138)

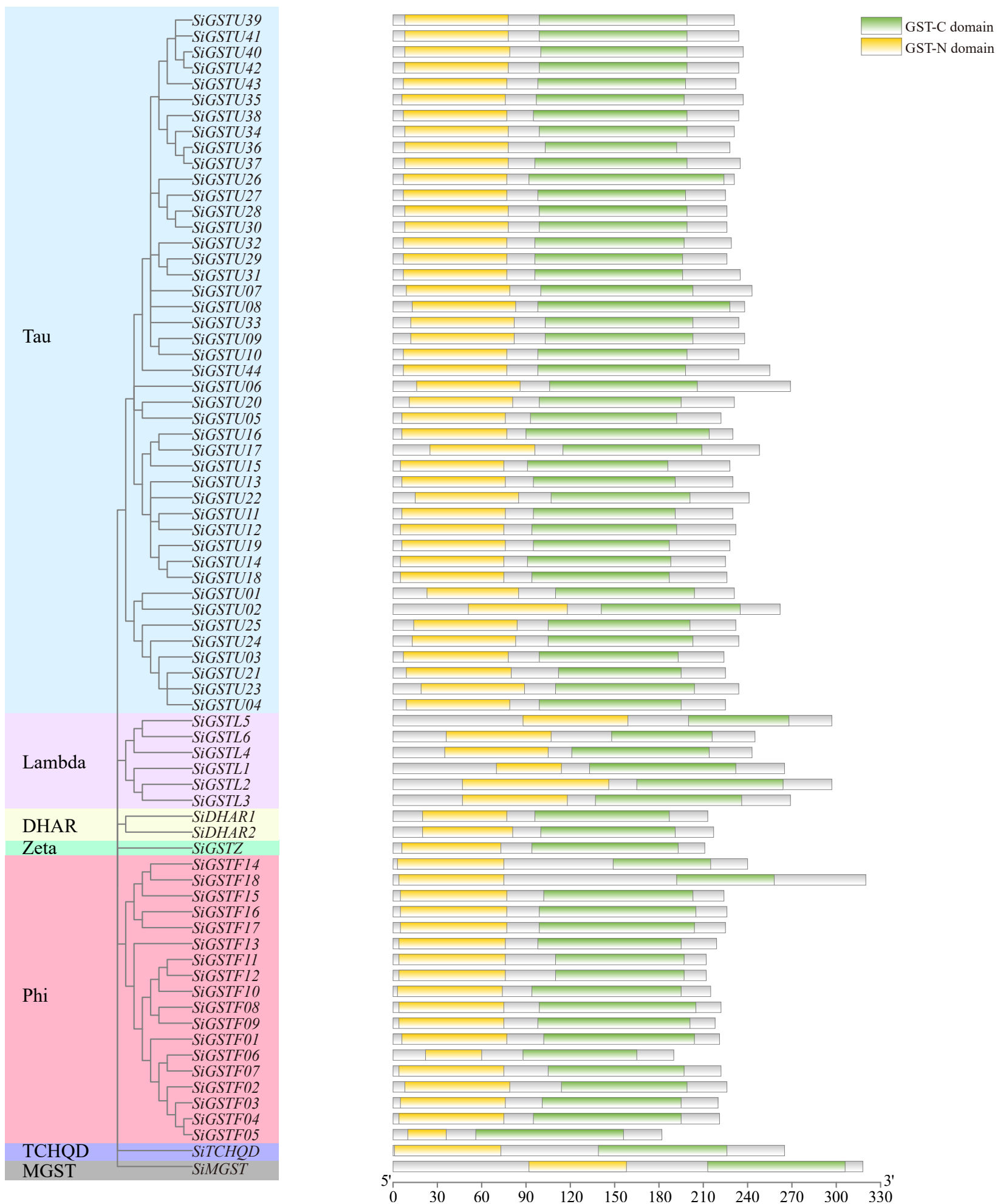

**Figure S1.** Conserved domain of foxtail millet *GSTs*.

Supplement: Supplementary file 1 [file plants-12-01138-s001.zip › Figure S1. Conserved domain of foxtail millet GSTs.pdf]

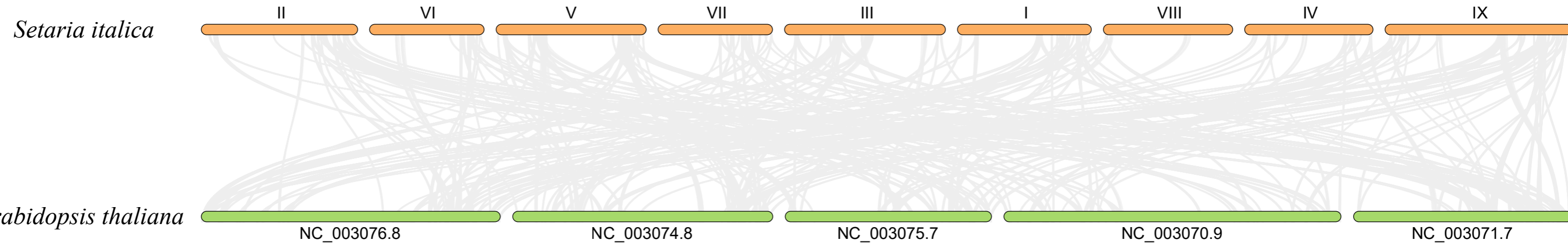

**Figure S2.** Collinearity analysis of *GSTs* in *Setaria italica* and *Arabidopsis thaliana*.

Supplement: Supplementary file 1 [file plants-12-01138-s001.zip › Figure S2. Collinearity analysis of GSTs in Setaria italica and Arabidopsis thaliana.pdf]

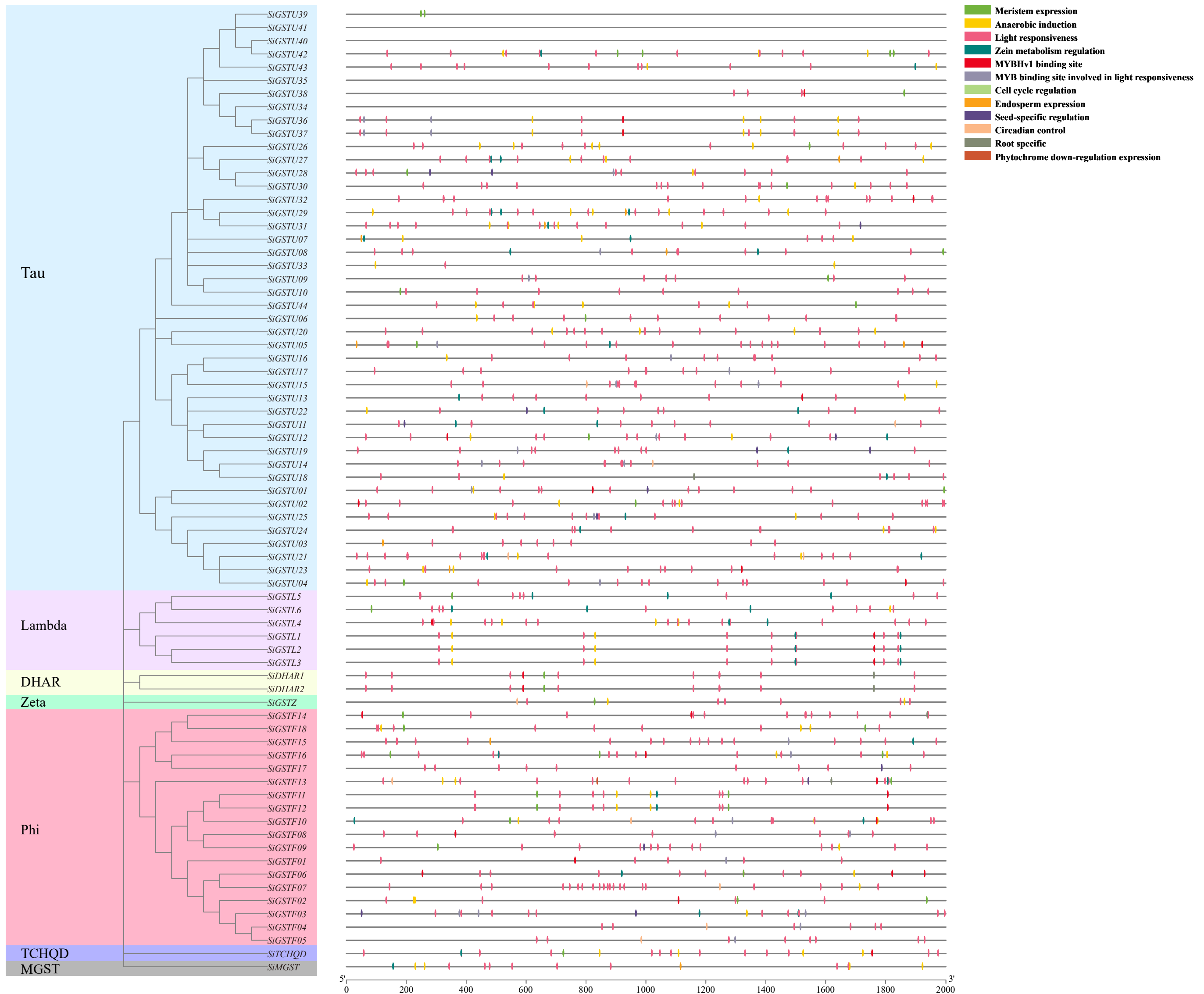

**Figure S4.** Predicted *cis*-acting elements in *SiGST* promoter of foxtail millet.

Supplement: Supplementary file 1 [file plants-12-01138-s001.zip › Figure S4. Predicted cis-acting elements in SiGST promoter of foxtail millet.pdf]
